# Effect of Triblock Copolymer on Carbon-Based Boron Nitride Whiskers for Efficient CO_2_ Adsorption

**DOI:** 10.3390/polym11050913

**Published:** 2019-05-21

**Authors:** Urooj Kamran, Kyong Yop Rhee, Soo-Jin Park

**Affiliations:** 1 Department of Chemistry, Inha University, 100 Inharo, Incheon 22212, Korea; malikurooj9@gmail.com; 2 Department of Mechanical Engineering, College of Engineering, Kyung Hee University, Yongin 17104, Korea

**Keywords:** structure directing approach, microporous boron nitride carbons, CO_2_ adsorption, gas selectivity

## Abstract

Herein, we investigated novel carbon-containing P123 copolymer-activated boron nitride whiskers (P123-CBNW) fabricated via a structure directing approach followed by a single-step heat treatment under N_2_. The resulting materials were found to be highly micro- and mesoporous. The influence of the activating agent (P123 copolymer) on the CO_2_ adsorption efficiency was determined. The prepared samples possessed high specific surface areas (594–1732 m^2^/g) and micropore volumes (0.258–0.672 cm^3^/g). The maximum CO_2_ uptakes of the prepared adsorbents were in the range 136–308 mg/g (3.09–7.01 mmol/g) at 273 K and 1 bar and 97–114 mg/g (2.22–4.62 mmol/g) in the following order: CBNW < P123-CBNW3 < P123-CBNW2 < P123-CBNW1 < P123-CBNW0.5. The isosteric heat of adsorption values (∆*Q*_st_) were found to be 33.7–43.7 kJ/mol, demonstrating the physisorption nature of the CO_2_ adsorption. Extensive analysis revealed that the presence of carbon, the high specific surface area, the high microporosity, and the chemical structural defects within the adsorbents are responsible for raising the CO_2_ adsorption ability and the selectivity over N_2_ gas. The fabricated adsorbents show excellent regeneration ability after several repeated adsorption cycles, making the prepared adsorbents promising candidates for gas storage applications.

## 1. Introduction

Carbon dioxide (CO_2_) emission is an issue of major concern because CO_2_ is a primary anthropogenic cause of environment variation and global warming. The International Monetary Fund (IMF) reported that up to 36 billion tons, considered an unacceptable level, of CO_2_ are released all over the world, harshly influencing human health, the ecosystem, as well as gross domestic products [1]. The common sources of CO_2_ emissions are coal fired plants and the combustion of fossil fuels include petroleum and mineral-coal, and methane gas generated from factories and the transportation and energy sectors [2,3,4,5]. Fossil fuels have a major impact on the increased CO_2_ levels, up to 406.4 ppm, which is directly responsible for further exacerbating the greenhouse effect [6]. For this reason, carbon capture and storage (CSS) is considered to be a very efficient approach to lower the levels of environmental CO_2_. While various defensive and remedial approaches to handle CO_2_ emissions are available, such as cryogenic distillation, liquid scrubbing, membrane separation, and conventional temperature or pressure swing (TSA or PSA) [7], these techniques have some drawbacks such as high energy consumption, greater processing time, and high cost [8,9]. At the commercial scale, traditional methods, such as wet scrubbing, utilize liquid amine-based materials for CO_2_ storage. Such methods possess some demerits including greater energy and cost consumption, equipment rusting, the toxic nature of amines and their byproducts, and loss of liquid from instruments [10,11]. Therefore, CO_2_ uptake by adsorption on various porous adsorbents, like covalent and porous organic polymers (COPs and POPs) [12,13,14], hexagonal/porous boron nitride (h-BN) [15,16], silica [17,18], polymeric materials [19], nitrogen doped carbon compounds [20,21], metal–organic frameworks (MOFs) [22,23], and metal oxide based carbonaceous materials [24,25,26], have been introduced as alternatives.

Among these adsorbents, porous boron nitride compounds (BN) seem to be the most effective because of their identical physiochemical characteristics, including high thermal and chemical durability and conductivity, low density, tunable high specific surface area (almost identical to activated carbon), few morphological defects, and possession of higher ordered chemistry as compared to carbonaceous adsorbents due to the existence of polar B–N bonds [27,28,29,30,31,32]. These advantageous properties lend porous BN to be favorable for applications in various areas, such as for gas adsorption of H_2_ [33], removal of organic pollutants and dyes from water [34], and use as a catalytic support. For instance, a previously fabricated porous BN material capable of CO_2_ uptake of up to 0.6 mmol/g was reported by Sofia et al. while h-BN synthesized by Chen et al. showed CO_2_ capture of up to 3.74 mmol/g at 298 K. Furthermore, Xiao et al. demonstrated CO_2_ uptake of about 0.45 mmol/g at 298 K by layered h-BN sheets [29], while CO_2_ adsorption of approximately 1.1 mmol/g was exhibited by BN pellets at 298 K [35]. On the basis of previous reports, we can conclude that CO_2_ capture by porous BN materials is relatively low. Therefore, future research in this area should focus on the development of porous BN materials that show greater CO_2_ adsorption ability. An efficient way to synthesize porous BN materials involves the decomposition reaction of boron and nitrogen reagents in the presence of additives for the induction of porosity and for structure directing purposes (CTAB and P123 copolymer) [36,37].

The research presented herein focuses on the development of unique carbon containing highly porous activated BN whiskers (CBNW) that exhibit high CO_2_ uptake ability. The activated porous CBNW were fabricated in the presence of triblock copolymer P123 [polyethylene glycol)-block-polypropylene glycol)-block-poly(ethylene glycol)] as a structure directing agent via structure directing methodology. The influence of P123 copolymer on the specific surface area and porosity of the synthetic materials and the resulting effect on the CO_2_ adsorption capacity of the materials were evaluated. The results show that a specified concentration of P123 copolymer in CBNW proved very efficient in improving CO_2_ uptake by increasing the microporosity and specific surface area of the material.

## 2. Experimental

### 2.1. Materials

Boric acid (H_3_BO_3_) was purchased from Duksan pure chemicals, Ansan, South Korea. Melamine (C_3_N_6_H_6_) was bought from Sigma Aldrich, St. Louis, MO, USA. Triblock copolymer P123 [polyethylene glycol)-block-polypropylene glycol)-block-poly(ethylene glycol)] with an average molecular weight of approximately 5800 was purchased from Sigma Aldrich, Riedstr, Germany. HNO_3_ (60% purity) was purchased from DAEJUNG KOSDAQ, Siheung-si, South Korea. All these chemicals were used as received without further purification.

### 2.2. Synthesis of P123 Activated Porous Carbon Containing BN Whiskers (P123-CBNW)

**Figure 1:**
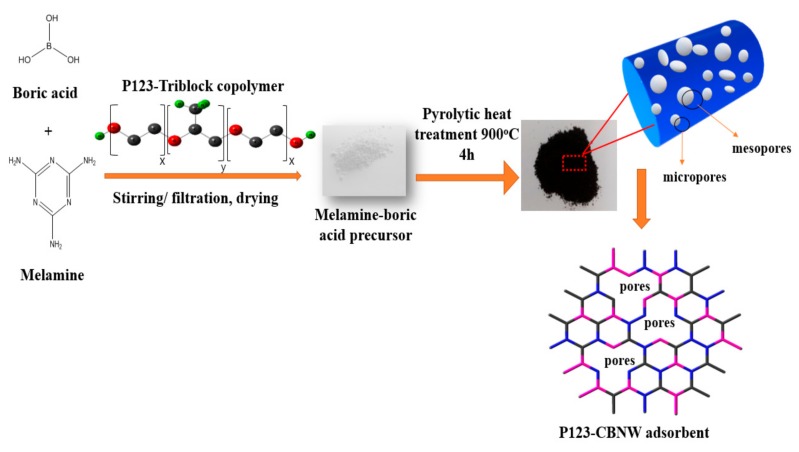
Schematic illustration of fabrication of P123 activated carbon containing porous boron nitride (BN) whiskers.

A brief schematic layout of the adsorbent synthesis is shown in Figure 1. First, 3.73 g of H_3_BO_3_ was dissolved in 250 mL of distilled water, then melamine (3.78 g) was slowly added with vigorous stirring. The pH of the mixture was kept at approximately pH 6 with 0.1 M HNO_3_. Next, 5 g of P123 copolymer was added to the reaction mixture at 90 °C under continuous stirring to produce a uniform solution. This mixture was allowed to react at the same conditions for 6 h after which the mixture was cooled to ambient temperature and the white product was collected by vacuum filtration and washed with cold distilled water. After drying at 100 °C for 8 h, the prepared powder was activated by pyrolytic treatment; in this case the white precipitates were calcined at low temperature (900 °C) for 4 h under N_2_ atmosphere with a flow rate of 150 mL/min to control carbon incorporation. The prepared product was abbreviated as P123-CBNW1. Other samples were prepared under the same conditions with half (2.5 g), double (10 g), and triple (15 g) the amount of P123 copolymer, designated as P123-CBNW0.5, P123-CBNW2, and P123-CBNW3, respectively, while the product fabricated without P123 copolymer was denoted as CBNW.

### 2.3. Characterization

The size and morphological analysis of the prepared materials was performed by high resolution scanning electron microscopy (HR-SEM; Model-SU8010, Hitachi Co. Ltd., Tokyo, Japan) at 1 kV and by transmission electron microscopy (TEM, JEM-2100F, JEOL, Tokyo, Japan). The thermal stability was assessed by thermo-gravimetric analysis (TGA; Model TG-209F3, NETZSCH, Bavarian, Germany). The functional group predictions were confirmed by Fourier transform infrared vacuum spectrometry (FTIR, Vertex- 80 V, Bruker, Billerica, MA, USA) wherein the spectra were obtained in the scanning range of 4000–400 cm^−1^. The X-ray diffraction peaks were obtained in the range of 2 theta angle (8–80°) using powder X-ray diffractometry (XRD, D-2 Phaser, Bruker Co, Panalytical Incorporated, Netherland). Elemental analysis was performed by Elemental-analyzer EA1112 (ThermoFisher scientific, Seoul, Korea). The surface properties were investigated by X-ray photoelectron spectroscopy (XPS, K-α, VG Scientific Co., Waltham, MA, USA) with monochromatic Mg Kα X-rays running at 150 W. Furthermore, the specific surface area, pore size distribution, and diameter of materials was obtained by N_2_ gas adsorption/desorption analysis using the instrument Belsorp Max (BEL-Japan). The target gas (CO_2_) capturing ability of the materials at different temperatures (273 K, 283 K, and 298 K) were also assessed with the Belsorp Max system (BEL-Japan Inc.). Prior to gas adsorption experiments, residual moisture in all samples was removed by degassing at 200 °C under vacuum for 6 h.

## 3. Results and Discussions

### 3.1. Structural and Morphological Analysis

**Figure 2:**
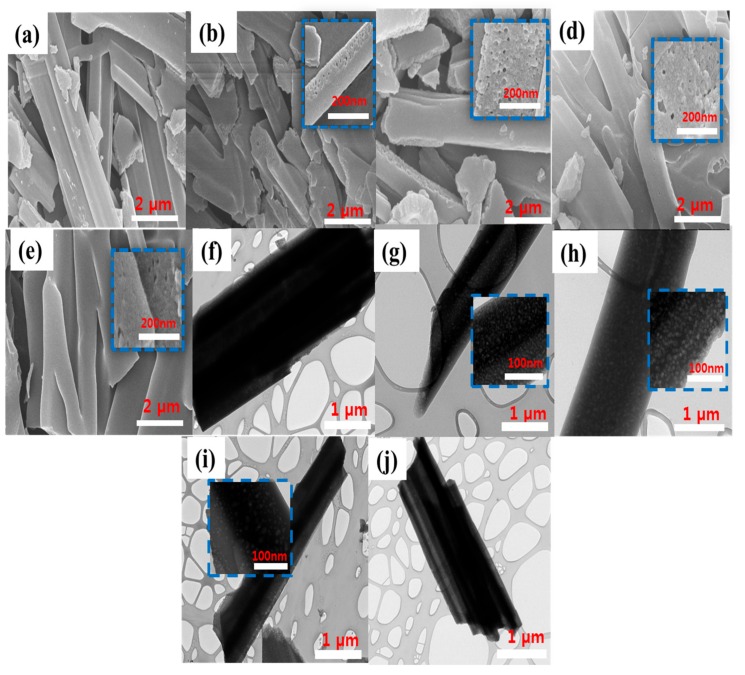
HR-SEM micrographs of samples (**a**) CBNW, (**b**) P123-CBNW0.5, (**c**) P123-CBNW1, (**d**) P123-CBNW2, and (**e**) P123-CBNW3. TEM images of (**f**) CBNW, (**g**) P123-CBNW0.5, (**h**) P123-CBNW1, (**i**) P123-CBNW2, and (**j**) P123-CBNW3. Insets show the high resolution photographs of the adsorbent surface.

The morphology of the prepared adsorbents was studied using HR-SEM and TEM at different micrometer and nanometer magnifications. The HR-SEM images in Figure 2a–e shows that CBNW and P123-CBNW samples exhibit disordered whisker-like non-uniform structures [38]. The TEM micrograph in Figure 2f shows the CBNW sample possessed minimum porosity; however, Figure 2g–h shows the poor crystalline nature of the adsorbents which is congruent with the XRD results. The insets indicate the porosity in P123-CBNW adsorbents introduced with P123-copolymer. The minimum size of the prepared samples was 100 nm. This type of disordered structure contributes to the porosity of the P123-CBNW materials [16]. The inset images show a close view of the material surface (200 nm resolution) displaying the highly porous nature generated by the activating agent during heat treatment. This is expected to play a significant role in enhancing the CO_2_ adsorption ability of the material [39].

**Figure 3:**
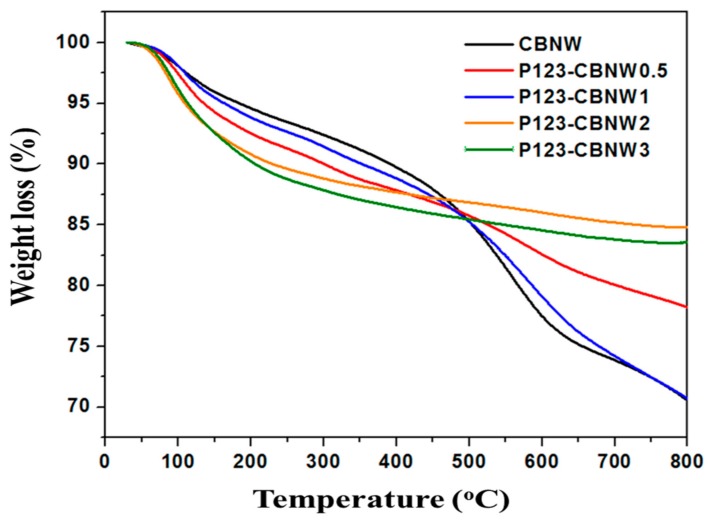
TGA curves of prepared samples in an air atmosphere (10 °C/min).

Additionally, TGA of prepared adsorbents was performed in an air atmosphere (rate = 10 °C/min) in order to determine the thermal stability and carbon content of the materials (Figure 3). The initial mass loss (5%) for all adsorbents below 106 °C is due to the elimination of residual solvent and moisture adsorbed on the material surface. It was noted that mass loss from 150–800 °C (for samples CBNW, P123-CBNW0.5, P123-CBNW1) and from 150–500 °C (for samples P123-CBNW2, P123-CBNW3) indicated the incorporation of carbon content within the samples, which undergoes combustion on raising the temperature. This weight loss can also be attributed to the removal of oxygen functionalities from the materials [40,41]. However, P123-CBNW2 and P123-CBNW3 undergoes stabilization above 500 °C.

**Figure 4:**
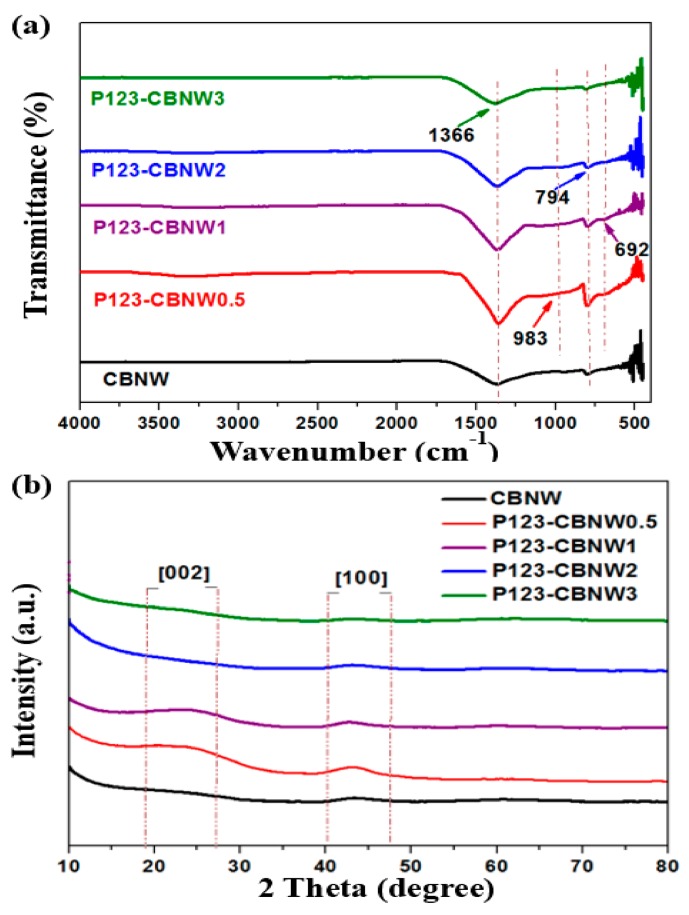
(**a**) FTIR spectra and (**b**) XRD patterns of CBNW, P123-CBNW0.5, P123-CBNW1, P123-CBNW2 and P123-CBNW3 samples.

The FTIR spectra in Figure 4a indicate the presence of functional groups and the nature of the bonds found within the prepared samples. The sharp shoulder at 1366 cm^−1^ and small peak at 794 cm^−1^ are attributed to two types of B–N stretching; the former value indicates in-plane B–N transverse stretching and the latter is indicative of above plane B–N–B bonds [42,43]. However, in P123 copolymer activated products the additional small shoulders at 983 and 692 cm^−1^ correspond to C–N, C–H, and B–N–O bonds, respectively [44,45,46]. The structure of the samples was further analyzed by XRD analysis. As mentioned in Figure 4b, an indiscernible peak appearing at 23° with low intensity shows plan phases of [002], indicating the existence of carbonaceous content in the prepared materials. Other peaks, at 43° with a phase index of [100] and at 61°, were attributed to poor crystallinity and defective structure, as confirmed by HR-SEM and TEM micrographs. These defects were due to the nanometer size of the whiskers [38] resulting in a turbostratic morphology, a type of shape found among hexagonal and amorphous materials [47,48].

The results of the elemental analysis are presented in Table 1. It was observed that carbon content increased from 14.99% (P123-CBNW0.5) to 25.12% (P123-CBNW3) due to the increasing amount of P123 copolymer.

**Figure 5:**
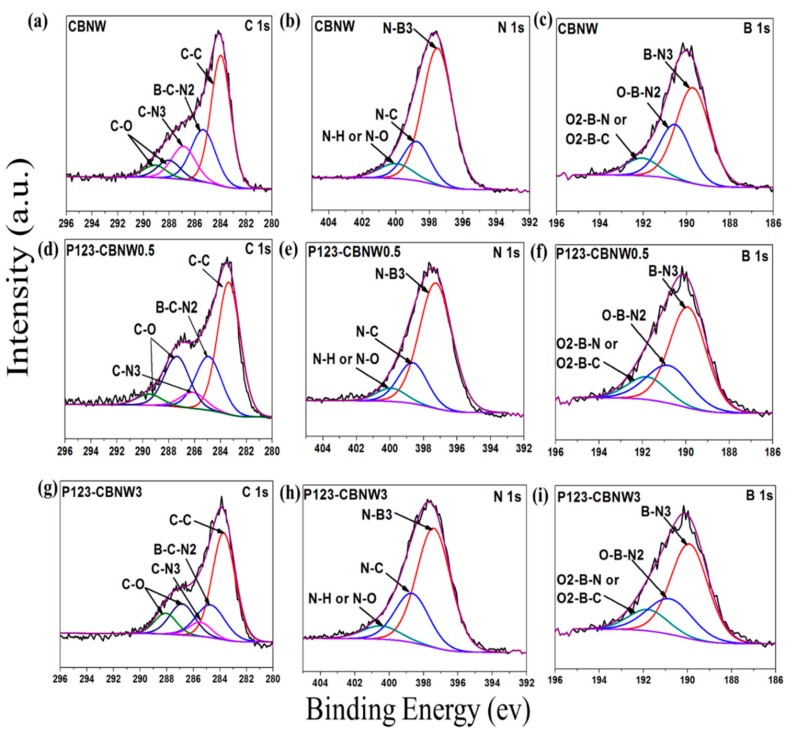
XPS scan of C1s (**a,d,g**), B1s (**b,e,h**), and N1s (**c,f,i**) of CBNW, P123-CBNW0.5 and P123-CBNW3 respectively.

The chemical environment of the atoms above the surface of the samples was characterized by XPS. In Figure 5a,d,g), the C1s deconvoluted scans of CBNW, P123-CBNW0.5, and P123-CBNW3 presented five binding energies. The high intensity peak at 283 eV in C1s is due to C–C bonds while the binding energies at around 285 and 286 eV correspond to the B–C–N2 and C–N3 bonds. The two remaining low intensity peaks in the region of 286–289 eV correspond to C–O species [49,50,51]. The scans of B1s (Figure 5b,e,h) demonstrated the deconvolution in to three main species; the first two relative peaks at 189 and 190 eV indicated B–N3 and O–B–N2 type binding. The other peak at approximately 192 eV corresponds to O2–B–N or O2–B–C species [52,53]. Furthermore, the N1s spectra of all adsorbents mentioned in Figure 5c,f,i exhibited a main peak at 397 eV corresponding to N–B3 type bonds. The remaining peak at 398 eV is attributed to N–C binding, while signals at 399 eV originate from N–H/N–O type bonding [54]. Therefore, the existence of C–C, C–N, and B–C–N bonds confirmed the presence of carbon in the boron nitride whiskers.

### 3.2. Textural Analysis

**Figure 6:**
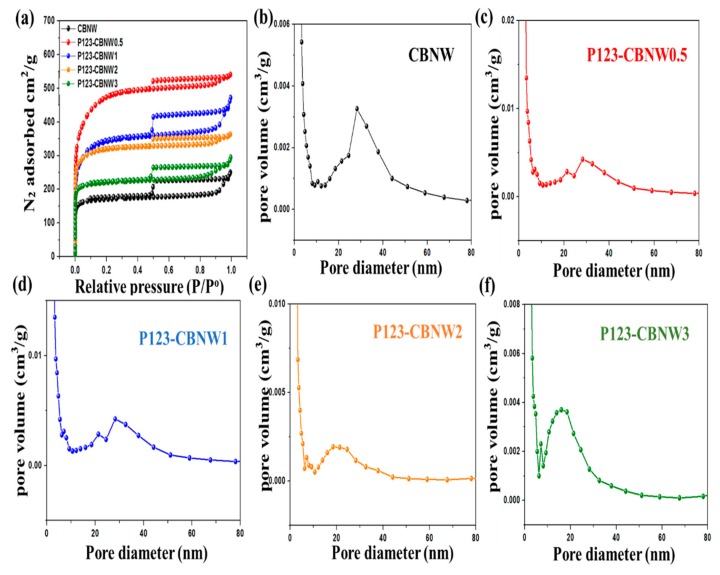
(**a**) N_2_ adsorption-desorption isotherms of prepared samples at 77 K, (**b–f**) non-local density functional theory equation (NLDFT)-pore size distribution plots of respective samples.

The textural analyses of CBNW and P123 copolymer activated adsorbents were obtained by nitrogen adsorption/desorption isotherms at 77 K. Prior to measurement, samples were degassed at 200 °C for 6 h under high vacuum conditions. Figure 6 illustrates the resulting isotherms and pore size distributions obtained by the non-local density functional theory equation (NLDFT). The shape of the isotherm indicated a IV type structure, which confirmed the existence of micro- and mesoporous structures that are linked via capillary condensation. 

The calculated numerical parameters, including specific surface area, total pore volume, micropore volume, and mesopore volume, are summarized in Table 2. It was noted that a minimum concentration of P123 copolymer plays a vital role in increasing the specific surface area of the fabricated materials (CBNW = 594 m^2^/g, P123-CBNW0.5 = 1732 m^2^/g). However, it was observed that a greater increase in the ratio of P123 copolymer lead to a decrease in specific surface area from 1281 m^2^/g (P123-CBNW1) to 764 m^2^/g (P123-CBNW3); concurrently, pore volume also declined due to inaccessibility of the pores to N_2_ due to blockage by excess activating agent (P123 copolymer) [55].

The maximum recorded specific surface area is much higher than that of the previously synthesized porous BN material (817 m^2^/g) [56]. The highest microporosity (0.67 cm^2^/g) was seen for P123-CBNW0.5 and it decreased as the activating agent ratio increased (P123-CBNW3 = 0.33 cm^2^/g) [57,58]. The pore volumes of prepared specimens were calculated by the Dubinin Radushkevich equation (Equation 1).

(1) $$W=W_{o}\exp\;\left[-B\left(\frac{T}{\beta }\right)log^{2}\left(\frac{p}{p_{o}}\right)\right]$$

Therefore, it can be summarized that the introduction of a quantified amount of P123 copolymer during fabrication is invaluable to the attenuation of the porous morphology and specific surface area of the resulting materials [59].

### 3.3. Gas Capture Analysis

**Figure 7:**
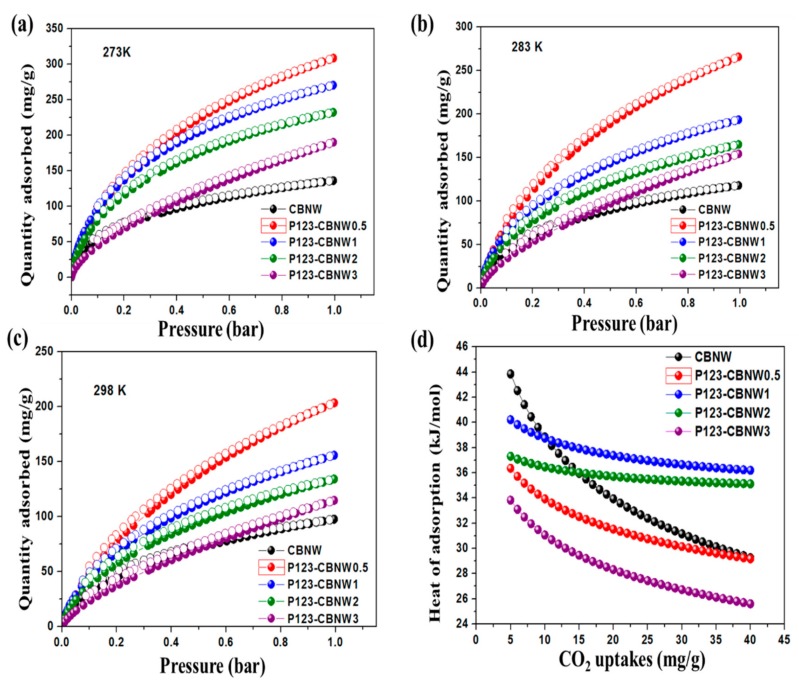
Adsorption, desorption isotherm of CO_2_ on prepared adsorbent materials at 273 K (**a**), 283 K (**b**), 298 K (**c**) and heat of adsorption of all materials (**d**).

The fabricated CBNW and P123-CBNW materials possessed higher specific surface areas and different micro- and mesoporous structures with various pore sizes. These properties enable us to evaluate their efficiency of CO_2_ uptake. Figure 7a–c presents the CO_2_ adsorption/desorption isotherms of samples collected at 273, 283, and 298 K at 1 bar while whole adsorption values are summarized in Table 3. CBNW shows a CO_2_ adsorption capacity of 136.2 mg/g at 273 K which increased with the addition of P123 copolymer (308.7 mg/g for P123-CBNW0.5) due to the high specific surface area, greater total pore volume, and increased microporosity of the polymer modified sample. In contrast, CO_2_ capture ability dropped from 270.5 mg/g (P123-CBNW1) to 190.2 mg/g (P123-CBNW3) with the continued increase in P123 copolymer. This decline in gas storage capacity occurred because of the following reasons: (1) decrease in micropore volume, and (2) lower specific surface area [35]. When considering the influence of temperature on CO_2_ uptake, it was observed that at 298 K, a clear decline in gas uptake occurred plateauing at 97.6, 203.6, 155.8, 134.2, and 114.8 mg/g for CBNW, P123-CBNW0.5, P123-CBNW1, P123-CBNW2, and P123-CBNW3, respectively. This trend depicts an exothermic adsorption phenomenon, wherein the CO_2_ capturing ability is inversely related to the adsorption temperature [60,61].

It is well-known that the important challenge is to adsorb CO_2_ gas at low pressure, however the high porous nature and functionality above the adsorbents provide enough active gas capturing centers to take acidic CO_2_ at low pressure (1 bar). In order to clearly understand the mechanism and reasons by which P123-CBNW adsorbents shows higher adsorption ability, it can be noted that the following two features attributed to the above mentioned results; (1) the porous morphology possesses strong merits for higher capture of CO_2_ molecules, however mesoporosity provides little resistance for adsorption of CO_2_ molecules, on the other hand higher microporosity provides a greater number of active sites for capturing CO_2_ easily. (2) the dual functionality –NH_2_ (attached to carbon) and –OH (attached to boron) also able the CO_2_ adsorption ability to be enhanced. The physiosorption of CO2 in-plane sites (functional groups) of adsorbents extensively based on vander waal interaction, that existed on the top sites of boron or nitrogen atoms as well as onto the bridges sites of B–N linkage [62,63].

In order to further investigate the nature of the adsorption process and the interaction between CO_2_ and the adsorbent materials, the Clausius–Clapeyron equation (Equation (2)) was applied to gas adsorption/desorption isotherms obtained at 273 and 298 K to calculate their isosteric heat of adsorption (∆*H*_ads_/∆*Q*_st_) [64].

(2) $$Q_{st}=R\;[\partial ln\frac{P}{\partial \left(\frac{1}{T\;}\right)}]\;\theta$$

While, *P* is pressure, *R* denotes real gas constant, *T* is adsorption temperature, and *θ* indicates the fraction of adsorbed sites. As presented in Figure 7d and Table 3, the calculated *Q*_st_ values determined for CO_2_ were 43.7, 36.7, 40.4, 37.6, and 33.7 kJ/mol for 5 mg of CBNW, P123-CBNW0.5, P123-CBNW1, P123-CBNW2, and P123-CBNW3, respectively. These small values correspond to a physisorption type of CO_2_ capture for the aforementioned adsorbent materials. As the CO_2_ adsorption capacity increased, the heat of adsorption declined due to the heterogeneous nature of the adsorption sites [16].

**Figure 8:**
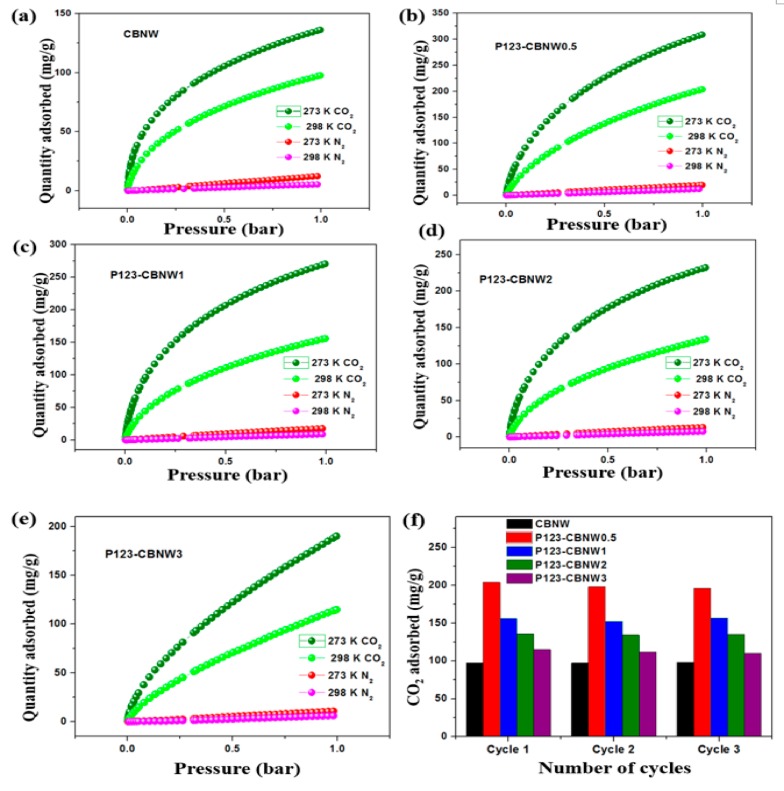
CO_2_ and N_2_ adsorption isotherm plots of CBNW (**a**), P123-CBNW0.5 (**b**), P123-CBNW1 (**c**), P123-CBNW2 (**d**), P123-CBNW3 (**e**), at 273 and 298 K for selective adsorption analysis, and (**f**) adsorption–desorption cycles for adsorbents at 298 K.

While selecting effective adsorbents for maximum CO_2_ capturing capacity, we also considered the selectivity over other gases such as N_2_ and CH_4_. That is, if the adsorbents showed excellent capacity for the uptake of all tested gases simultaneously, they were considered unsuitable for CO_2_ capture. Herein, we also assessed the CO_2_/N_2_ selectivity of the prepared materials at 273 and 298 K (1 bar) as seen in Figure 8a–c. The N_2_ adsorption capacity was very low as compared to CO_2_ adsorption at both temperatures (at 1 bar). Both the ideal adsorbed solution theory (IAST) method and Henry’s law were applied to find the CO_2_/N_2_ selectivity by adsorbents. The equilibrium selectivity of CO_2_ gas (X) over N_2_ gas (Y) on the adsorbents was calculated by the ratio of the initial slopes obtained by Henry’s law as shown in following equation,

(3) $$S_{x,y}=\frac{K_{x}}{K_{y}}$$

**Figure 9:**
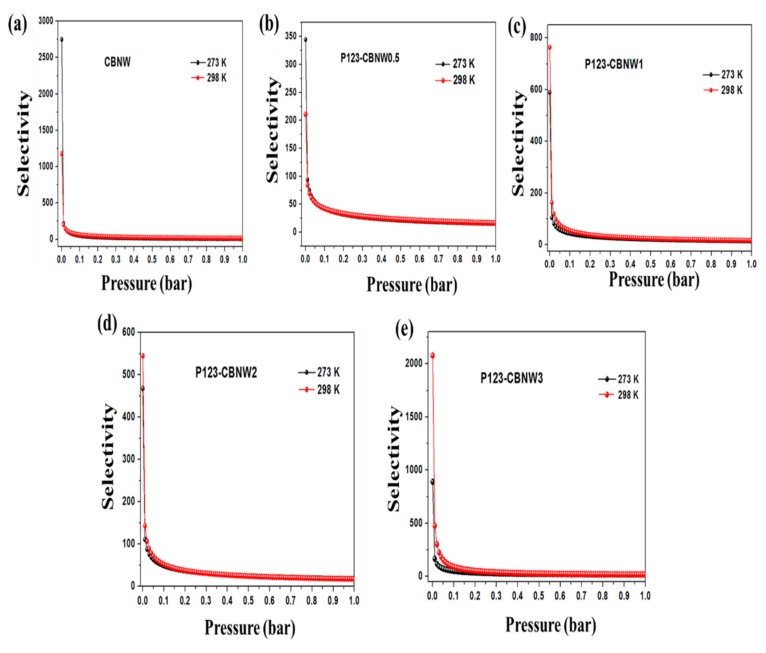
CO_2_/N_2_ selectivity using ideal adsorbed solution theory (IAST) method of CBNW (**a**), P123-CBNW0.5 (**b**), P123-CBNW1 (**c**), P123-CBNW2 (**d**), and P123-CBNW3 (**e**) at 273 and 298 K.

*K_x_* and *K_y_* represent the slope obtained from the single isotherm of CO_2_ and N_2_ gas. However, IAST calculations was performed by fitting the specific model; in this case one site and double site Langmuir Freundlich models has been applied to compare the pure-adsorbent equilibrium data and identify the adsorption of gas mixture. The calculated adsorption selectivities at 1 bar by Henry’s law were 44.89 (CBNW), 37.81 (P123-CBNW0.5), 40.43 (P123-CBNW1), 43.91 (P123-CBNW2), and 41.13 (P123-CBNW3) at 273 K and 52.82 (CBNW), 37.92 (P123-CBNW0.5), 46.42 (P123-CBNW1), 43.23 (P123-CBNW2), and 64.58 (P123-CBNW3) at 298 K for CO_2_ over N_2_. However, by the IAST method the investigated selectivities were 118.97, 65.07, 70.3, 76, 95.12 at 273 K (1 bar) and 117.03, 61.75, 98.69, 89.13, 224.97 at 298 K (1 bar) for samples CBNW, P123-CBNW0.5, P123-CBNW1, P123-CBNW2, and P123-CBNW3 respectively. The IAST plots are presented in Figure 9a–e. The higher CO_2_ gas selectivity of the carbon containing BN samples was due to the physisorption attraction of the polar B–N bond towards the acidic CO_2_ gas. This covalent polar bond and highly porous nature of the materials enhanced their affinity towards CO_2_; in contrast, other carbon-based materials did not exhibit the same abilities [65,66]. From an economical point of view, we also focused on the regenerative ability of the prepared adsorbents. In this study, we examined this aspect of the adsorbents by performing four repeated CO_2_ adsorption cycles at 298 K and 1 bar (Figure 8d). The results indicated that the prepared samples exhibited high potential for regeneration and recyclability, thus making the adsorbents promising candidates for gas storage applications.

## 4. Conclusions

In this work, we successfully fabricated highly microporous and mesoporous carbon containing P123-CBNW adsorbents. This was accomplished through a structure directed approach using boric acid and melamine as boron and nitrogen precursors along with a triblock copolymer as an activating agent followed by a single step pyrolytic heat treatment under a N_2_ atmosphere. The prepared P123-CBNW0.5 and P123-CBNW1 adsorbents exhibited high micro- and mesoporosity, structural chemical defects, and high specific surface areas (1732 and 1281 m^2^/g, respectively), which are responsible for the increase in CO_2_ adsorption ability (308 and 270 mg/g, respectively) as compared to CBNW (136 mg/g) at 273 K and 1 bar. All prepared materials exhibited high selectivity for CO_2_ over N_2_. The CO_2_ capture ability of the materials increased in the order of CBNW < P123-CBNW3 < P123-CBNW2 < P123-CBNW1 < P123-CBNw0.5 and the aforementioned samples possessed higher CO_2_ adsorption capacity than previously reported materials (Table 4). On the basis of our reported work, it was demonstrated that the adsorption ability can be increased by altering the microporosity and surface area of the adsorbents through the addition of a minor amount of activating agent. The adsorbents show excellent regeneration ability after several repeated adsorption cycles. Therefore, these reported materials prove to be very efficient for future gas storage applications.

## Figures and Tables

**Table 1:** Elemental analyses of the adsorbents fabricated.

Samples	Weight (%)		
	C ^a^	H ^b^	N ^c^
CBNW	12.59	2.61	29.62
P123-CBNW0.5	14.99	2.44	30.41
P123-CBNW1	16.45	2.81	29.65
P123-CBNW2	21.34	2.92	25.15
P123-CBNW3	25.12	3.01	26.54

^a^ Carbon contents. ^b^ Hydrogen contents. ^c^ Nitrogen contents.

**Table 2:** Textural properties of prepared materials from N_2_ isotherms at 77 K.

Materials	*S*_BET_^a^	*V*_total_^b^	*V*_meso_^c^	*V*_micro_^d^
CBNW	594	0.3829	0.1249	0.2580
P123-CBNW0.5	1732	0.8351	0.1628	0.6723
P123-CBNW1	1281	0.718	0.22	0.4960
P123-CBNW2	1128	0.5606	0.085	0.4756
P123-CBNW3	764	0.4488	0.1157	0.3331

^a^ Specific surface area (m^2^/g) obtained by Brunauer–Emmett–Teller (BET) method. ^b^ Total pore volume (cm^3^/g) calculated at *P/P0* = 0.99. ^c^ Mesopore volume (cm^3^/g) analyzed by Barrett-Joyner-Halenda (BJH) method. ^d^ Micropore volume (cm^3^/g) obtained by D-R equation.

**Table 3:** CO_2_ and N_2_ gas uptake by the prepared samples at different temperatures at 1 bar pressure.

Samples	CO_2_ Uptakes (mg/g)			*Q*_st_ for CO_2_ (kJ/mol)	N_2_ Uptakes (mg/g)	
	273 K	283 K	298 K		273 K	298 K
CBNW	136.2	118.1	97.6	43.8	12.3	5.3
P123-CBNW0.5	308.7	265.7	203.6	36.4	19.8	11.9
P123-CBNW1	270.5	193.5	155.8	40.2	17.6	8.8
P123-CBNW2	232.4	165.1	134.2	37.3	13.1	7.6
P123-CBNW3	190.2	154.3	114.8	33.8	10.7	6.1

**Table 4:** Comparative study of CO_2_ capture capacities of prepared sample with various adsorbents at 273 K and 1 bar.

Adsorbents	CO_2_ Uptakes (mmol/g)	References
P123-CBNW0.5	7.01	Present work
R-HAC 20	5.57	[67]
BCN (2:1)	5.50	[16]
NEPB-3UK	4.8	[68]
CF-850-act	4.41	[20]
PAN-PK	4.40	[69]
APAB-3	4.1	[70]
NHPC4	3.96	[71]
PCTP-2	3.45	[57]
SG–MOP-5	3.37	[72]
PHAP-2	2.37	[14]
